# Pulmonary vasoconstrictor action of KCNQ potassium channel blockers

**DOI:** 10.1186/1465-9921-7-31

**Published:** 2006-02-20

**Authors:** Shreena Joshi, Prabhu Balan, Alison M Gurney

**Affiliations:** 1 Department of Physiology & Pharmacology, University of Strathclyde, 27 Taylor street, Glasgow G4 0NR, UK; 2 Faculty of Life Sciences, University of Manchester, Floor 2, Core Technology Facility, University of Manchester, 46 Grafton Street, Manchester M13 9NT, UK

## Abstract

**Background:**

KCNQ channels have been widely studied in the nervous system, heart and inner ear, where they have important physiological functions. Recent reports indicate that KCNQ channels may also be expressed in portal vein where they are suggested to influence spontaneous contractile activity. The biophysical properties of K^+ ^currents mediated by KCNQ channels resemble a current underlying the resting K^+ ^conductance and resting potential of pulmonary artery smooth muscle cells. We therefore investigated a possible role of KCNQ channels in regulating the function of pulmonary arteries by determining the ability of the selective KCNQ channel blockers, linopirdine and XE991, to promote pulmonary vasoconstriction.

**Methods:**

The tension developed by rat and mouse intrapulmonary or mesenteric arteries was measured using small vessel myography. Contractile responses to linopirdine and XE991 were measured in intact and endothelium denuded vessels. Experiments were also carried out under conditions that prevent the contractile effects of nerve released noradrenaline or ATP, or block various Ca^2+ ^influx pathways, in order to investigate the mechanisms underlying contraction.

**Results:**

Linopirdine and XE991 both contracted rat and mouse pulmonary arteries but had little effect on mesenteric arteries. In each case the maximum contraction was almost as large as the response to 50 mM K^+^. Linopirdine had an EC_50 _of around 1 μM and XE991 was almost 10-fold more potent. Neither removal of the endothelium nor exposure to phentolamine or α,β-methylene ATP, to block α_1_-adrenoceptors or P2X receptors, respectively, affected the contraction. Contraction was abolished in Ca^2+^-free solution and in the presence of 1 μM nifedipine or 10 μM levcromakalim.

**Conclusion:**

The KCNQ channel blockers are potent and powerful constrictors of pulmonary arteries. This action may be selective for the pulmonary circulation as mesenteric arteries showed little response. The results imply that the drugs act directly on smooth muscle cells and contraction requires voltage-dependent Ca^2+ ^influx. It is concluded that the drugs probably act by blocking KCNQ channels in pulmonary artery myocytes, leading to membrane depolarization and Ca^2+ ^influx through L-type Ca^2+ ^channels. This implies a functional role for KCNQ channels in regulating the resting membrane potential of pulmonary artery myocytes.

## Background

KCNQ (Kv7) genes encode a family of voltage-gated K^+ ^channels with 6 membrane spanning domains and a single P-loop that forms the selectivity filter of the pore. Members of this channel family have been widely studied in the nervous system, heart and inner ear, where they have important physiological functions [1,2]. In the nervous system, KCNQ channels are thought to underlie the M-current, a non-inactivating, voltage-dependent K^+ ^current that plays a critical role in regulating neuronal excitability and action potential firing frequency [3]. It was originally thought that M-current is mediated by heteromultimeric channels formed by KCNQ2 and KCNQ3 subunits [4], but it may also require association with the regulatory, KCNE2 subunit [5]. Moreover, K^+ ^currents with characteristics similar to M-current can also be produced by homomultimers of KCNQ1, KCNQ2, KCNQ3, KCNQ4 and KCNQ5 [2], as well as heteromers of KCNQ3 and KCNQ5 [6,7]. Thus there may be significant molecular diversity in the composition of M-like currents in different cell types.

K^+ ^currents produced by the heterologous expression of different KCNQ genes in *Xenopus *oocytes or mammlian cell lines display distinct properties. In general though, they can be activated at rather negative membrane potentials, below -60 mV, are outwardly rectifying and show little or no inactivation [2]. Similar characteristics are displayed by a K^+ ^current found in pulmonary artery smooth muscle cells, which plays a key role in regulating the resting membrane potential [8]. Although part of this current has been proposed to be mediated by two-pore domain TASK channels [9], there is a residual component that remains to be identified. Its similarity to the M-current has led us to speculate that it might be mediated by KCNQ channels and that KCNQ channels might therefore play a role in regulating the resting membrane potential of pulmonary artery smooth muscle cells. Consistent with this idea, expression of KCNQ1 and the regulatory subunit, KCNE4, has been reported in lung [10,11]. Although not examined in pulmonary arteries, KCNQ1 transcripts have also been reported in the mouse portal vein [12], where there is evidence that they have a functional role in regulating spontaneous contractile activity [13].

Inhibition of the K^+ ^channels contributing to the resting membrane potential of pulmonary artery smooth muscle cells would cause membrane depolarisation. If the cells depolarise sufficiently to reach the threshold for activating L-type Ca^2+ ^channels, the result would be Ca^2+ ^influx and muscle contraction. The ability of inhibitors of KCNQ channels to induce pulmonary artery contraction would therefore provide support for a role of these channels in regulating the resting potential. Due to their selective inhibition of KCNQ channels, the drugs linopirdine and XE991 (a more potent analogue of linopirdine) have been used as markers of these channels [1,2]. Their effect on M-current is thought to be a major factor in the abilities of these drugs to stimulate the release of central neurotransmitters [14], an action that led to these drugs being considered as cognition enhancers and investigated for the treatment of Alzheimer's disease. Linopirdine inhibits native M-currents at low micromolar concentrations and is around 20-fold less potent at inhibiting other voltage-gated K^+ ^channels [15]. Linopirdine and XE991 inhibit heterologously expressed KCNQ channels with varying potency, depending on their subunit composition, but at concentrations effective at blocking KCNQ channels they are without effect on a number of other recombinant K^+ ^channels [2,4,16].

This study investigated the effects of the KCNQ channel blockers, linopirdine and XE991, on isolated rat and mouse pulmonary arteries, with the aim of determining whether or not KCNQ channels could potentially play a role in regulating the resting membrane potential and tone of pulmonary artery smooth muscle.

## Methods

This investigation was carried out under regulations dictated by the UK Scientific Procedures (Animals) Act 1986 and conforms to the *Guide for the Care and Use of Laboratory Animals *published by the National Institutes of Health (NIH Publication No. 85-23, revised 1996). Male Sprague Dawley rats (250–300 g) and BALB/c mice (4–5 weeks old) were killed by cervical dislocation. The lungs and mesentery were rapidly excised into physiological salt solution (PSS) containing (mM): NaCl 120; KCl 5; MgCl2 1; NaH2PO4 0.5; KH2PO4 0.5; glucose 10; CaCl_2 _1; pH 7.4. Intrapulmonary arteries and mesenteric arteries with external diameters of 300–400 μm (rats) or 100–200 μm (mice) were dissected free of connective tissue and mounted in a small vessel myograph (Danish Myotechnology). The vessels were bathed in PSS, continually aerated at 37°C. A basal tension of 5 mN (rat) or 4 mN (mouse) was applied and vessels allowed to equilibrate for 40 min, washing every 15 min, before beginning the experiment.

At the start of each experiment, vessels were exposed to 50 mM K^+ ^until a maximal contractile response was observed. This was done by replacing the PSS in the bath with PSS in which the K^+ ^concentration was increased to 50 mM by equimolar substitution of KCl for NaCl. The vessels were then washed until tension returned to baseline and the challenge with high K^+ ^was repeated until reproducible contractions were produced. Following washout and recovery of tension to baseline, the tissue was then exposed to increasing concentrations of linopirdine (1 nM – 100 μM) or XE991 (0.1 nM – 100 μM), applied in a cumulative manner with an interval of at least 10 min between additions. Contractile responses to these drugs were measured as a percentage of the response to 50 mM K^+ ^in each vessel. In some experiments the endothelium was removed by rubbing the lumen of the vessel with a human hair. The failure of acetylcholine (1 μM) to relax vessels pre-constricted with 1 μM phenylephrine was taken to indicate successful denudation. As relaxant responses were not always abolished by this procedure, experiments on endothelium-denuded vessels were also carried out in the presence of the nitric oxide synthase inhibitor N(G)-nitro-L-arginine methyl ester (L-NAME, 100 μM) and the cyclooxygenase inhibitor indomethacin (10 μM).

Rat intrapulmonary arteries were used to investigate the mechanism of constriction caused by the KCNQ channel blockers. The response to a single application of linopirdine (10 μM) or XE991 (1 μM) was first established. After washout and recovery to baseline tension, the tissue was then bathed for 20 min in Ca^2+^-free bath solution, which was identical to PSS but with CaCl_2 _omitted and 1 mM EGTA added. The vessels were then challenged again with linopirdine or XE991. To control for time-dependent changes in the response amplitude, in some experiments linopirdine or XE991 was first applied in Ca^2+^-free bath solution and the application repeated after returning to normal PSS. The effects of nifedipine (1 μM), levcromakalim (10 μM) and phentolamine (10 μM) on contractile responses to the KCNQ channel blockers were tested in a similar way. Vessels were challenged once with linopirdine (10 μM) or XE991 (1 μM) under control conditions and once in the presence of each of these drugs.

The dihydrochloride salts of linopirdine and XE991 were purchased from Tocris Bioscience. Stock solutions (10 mM) prepared in distilled water were stored as frozen aliquots and diluted to the final bath concentration in PSS. A few experiments used linopirdine from Sigma, stored as a 10 mM stock dissolved in dimethylsulphoxide. Nifedipine, levcromakalim, phentolamine hydrochloride, phenylephrine hydrochloride, acetylcholine chloride and ethylene glycol-bis(2-aminoethylether)-N,N,N',N'-tetraacetic acid (EGTA) were all from Sigma. Data are expressed as means ± S.E.M of n animals and compared using paired or unpaired Student's t test, as appropriate. Differences were considered statistically significant when p < 0.05.

## Results

### Pulmonary vasoconstriction by KCNQ channel blockers

**Figure 1:**
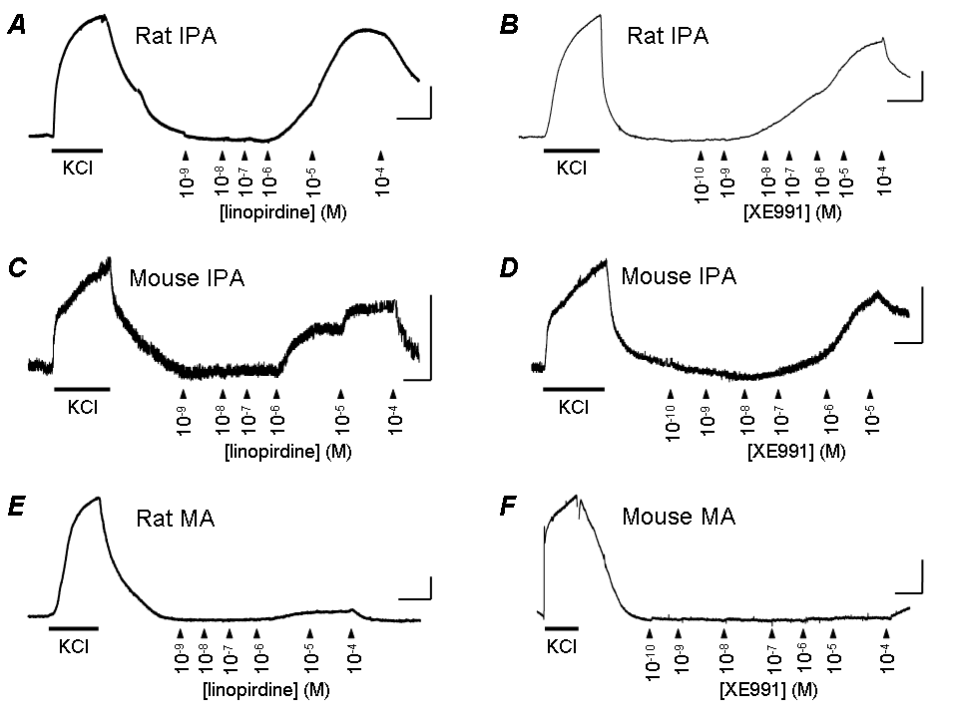
**KCNQ channel blockers constrict pulmonary, but not mesenteric artery**. Original records of tension developed by rat (*A*, *B*) and mouse (*C*, *D*) intrapulmonary artery (IPA) in the presence of 50 mM KCl or increasing concentrations of linopirdine or XE991, applied where indicated. Original records of tension developed by rat (*E*) and mouse (*F*) mesenteric arteries (MA) show responses to 50 mM KCl, but not linopirdine or XE991 applied at the same concentrations. Calibration bars are 1 mN (vertical) and 10 min (horizontal).

**Figure 2:**
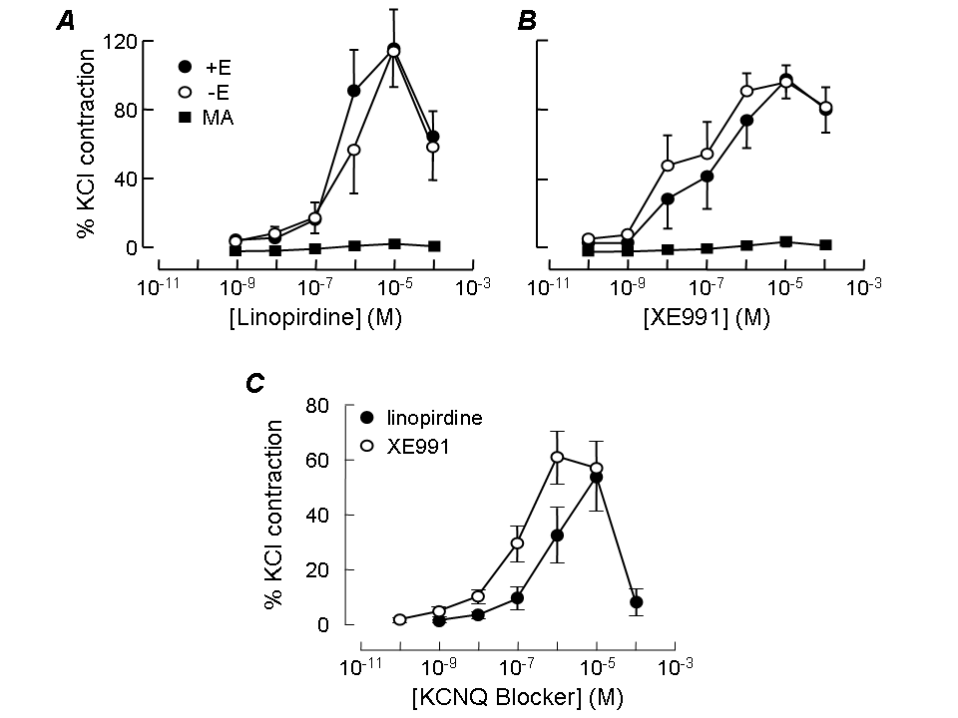
**Concentration-response curves for pulmonary artery constriction by KCNQ channel blockers**. Concentration dependence of contraction induced by linopirdine (*A*) or XE991 (*B*) in rat intrapulmonary arteries that were intact (+E) or denuded of endothelium (-E). Plots also show the mean responses of rat mesenteric arteries (MA) at the same drug concentrations. Each point represents the mean ± SEM of 5–6 experiments. *C*. Concentration dependence of contraction induced by linopirdine (n = 9) or XE991 (n = 11) in intact mouse intrapulmonary arteries. Responses were measured in each vessel as a percentage of the contractile response to 50 mM KCl.

**Figure 4:**
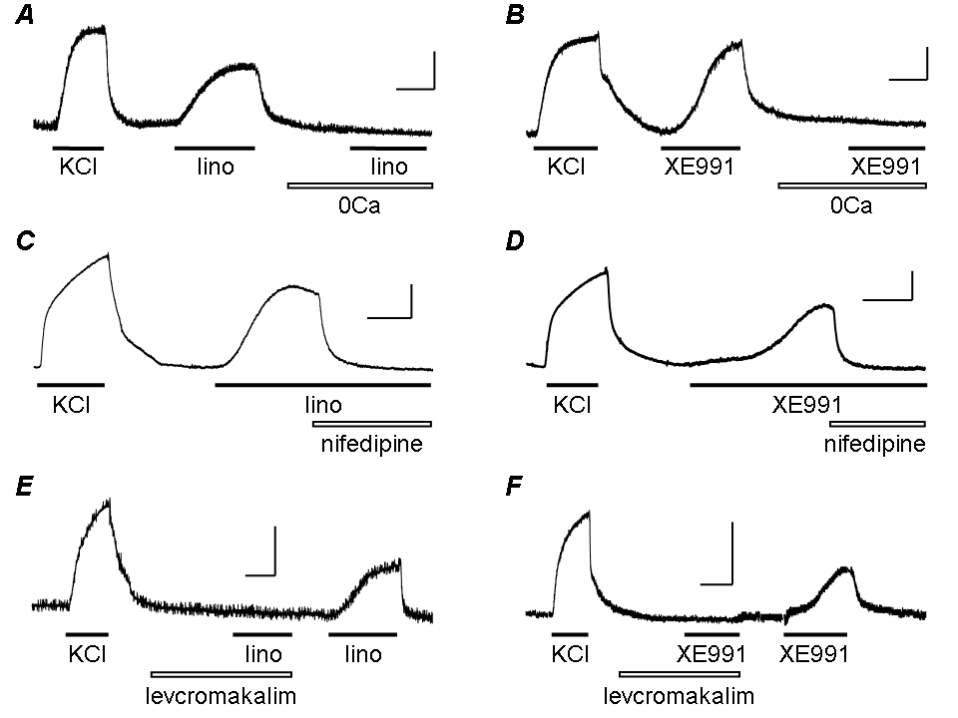
**Mechanism of contraction induced by KCNQ channel blockers**. Original records show contractile responses of rat intrapulmonary artery to 50 mM KCl, followed by 10 μM linopirdine (*A*, *C*, *E*) or 1 μM XE991 (*B*, *D*, *F*) under control conditions and in the presence of Ca^2+^-free (0Ca) bathing solution (*A*, *B*), 1 μM nifedipine (*C*, *D*) or 10 μM levcromakalim (*E*, *F*).

When applied to rat intrapulmonary arteries both linopirdine and XE991 produced concentration-dependent contraction with an amplitude that was often as large as the contraction induced by 50 mM K^+ ^(Fig 1A,B). Figure 2A shows the concentration-response curve for linopirdine, constructed from the mean data obtained from 6 vessels. Each increase in drug concentration was made after contraction had appeared to reach a steady level and tension was measured as the level reached immediately before addition of the higher concentration. It can be seen that the drug was maximally effective at 10 μM. When the data in Fig 1A was pooled with experiments in which vessels were challenged with a single application of 10 μM linopirdine, the mean contraction at this concentration had an amplitude 85 ± 12% (n = 21) of that induced by 50 mM K^+^. No further contraction was observed at higher linopirdine concentrations. In fact, upon addition of 100 μM linopirdine, vessels displayed relaxation (Fig. 1A) with tension returning around 30% towards the baseline (Fig. 2A). This precluded fitting the Hill equation to obtain the concentration producing 50% of the maximum response (EC_50_), which was instead estimated by interpolation of the concentration-response curves for each vessel tested. This gave EC_50 _= 1.3 ± 0.5 μM (n = 6) for linopirdine. XE991 was more potent than linopirdine, often producing substantial contractions below 10 nM (Fig. 1B). The EC_50_, estimated as above, was 0.4 ± 0.3 μM (n = 6). The maximal response was usually observed at 1 or 10 μM XE991, where it was nearly as large as the contractile response to 50 mM K^+ ^(Fig 2B). The mean amplitude of all contractions induced by 1 μM XE991 was 79 ± 8% (n = 21) of the response to K^+^. This was not significantly different from the maximum response to linopirdine. As seen with linopirdine, during cumulative applications of XE991 relaxation was sometimes observed at high concentrations (10 μM). The time course of the contractile responses to linopirdine and XE991 were somewhat variable. Most figures show traces in which the contractions developed over 15 min or so and recovered fully within around 30 min. In many tissues however, contraction took up to 30 min to develop, sometimes beginning after a substantial delay (e.g. Fig. 4D), and complete recovery often took over an hour after washing. This variable time course means that although, during experiments, contraction appeared to reach a steady level at each concentration, it may not always have developed fully at low concentrations (e.g. Fig. 1A,B,D), which could cause the EC_50 _values to be overestimated.

Both linopirdine and XE991 had similar effects on mouse intrapulmonary arteries (Fig. 1C,D). The concentration dependence of their actions on mouse vessels is shown in Fig. 2C. The EC_50 _for linopirdine, estimated as above, was 0.6 ± 0.2 μM (n = 9). The EC_50 _for XE991 was 0.12 ± 0.03 μM (n = 11), making it significantly more potent than linopirdine (p < 0.05). The drugs were maximally effective at the same concentrations observed in rat arteries and the maximal contractions of mouse pulmonary artery to the two drugs were not significantly different. Linopirdine (10 μM) induced a contraction amounting to 54 ± 12% (n = 9) of the response to 50 mM K^+ ^while the contraction induced by 1 μM XE991 was 61 ± 10% (n = 11) of the response to K^+^. Also as observed in rat, during cumulative applications a relaxation response to both drugs became apparent at the highest concentrations tested (Fig. 1C,D, Fig. 2C.

In contrast to the effects of linopirdine and XE991 on pulmonary arteries, neither drug had a significant effect on mesenteric arteries from the same animals. Original traces show little effect on rat (Fig. 1E) or mouse (Fig. 1F) mesenteric arteries, despite them contracting strongly in response to the application of 50 mM K^+^. The lack of effect is clear in the concentration-effect curves shown for rat in Fig. 2A,B, where responses in mesenteric artery are directly compared with intrapulmonary arteries of similar size.

### KCNQ channel blockers act directly on pulmonary artery smooth muscle

Both linopirdine and XE991 continued to constrict pulmonary arteries after endothelial function was blocked, by removing endothelium from the vessel lumen and inhibiting the release of nitric oxide and prostacyclin. In fact the concentration-response curves to linopirdine (Fig. 2A) and XE991 (Fig. 2B) were essentially unaltered in the absence of endothelium, indicating that the presence of endothelium is not needed for the drugs to cause contraction.

**Figure 3:**
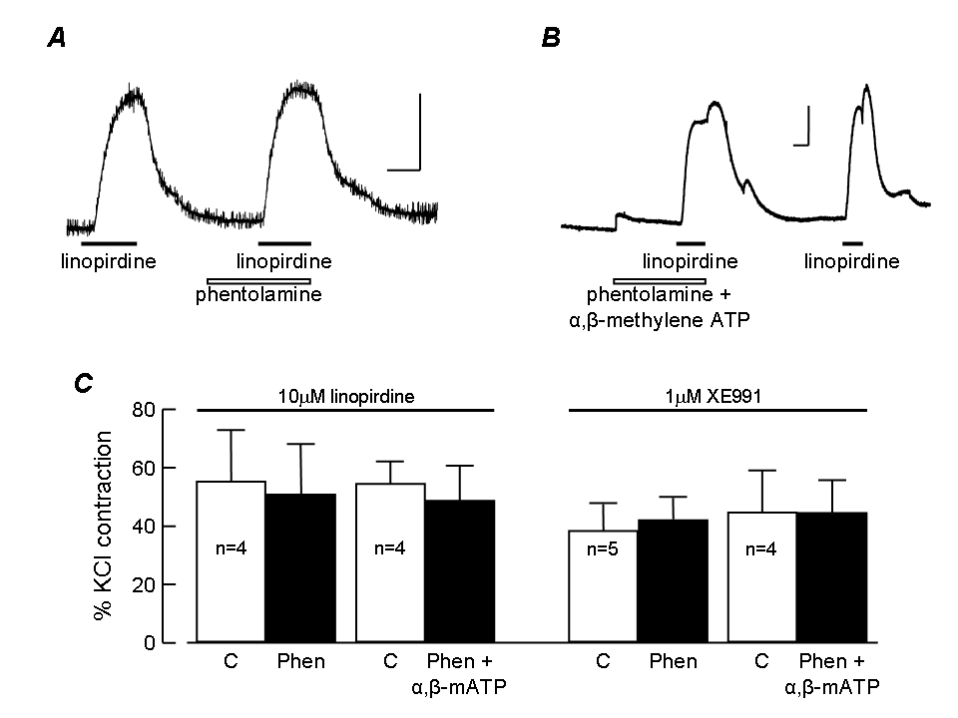
**Contraction is independent of nerve released noradrenaline or ATP**. *A*. Original record showing contraction of rat intrapulmonary artery to 10 μM linopirdine before and then during exposure to 10 μM phentolamine. *B*. Original record showing contraction of rat intrapulmonary artery to 10 μM linopirdine after exposing the tissue to 10 μM phentolamine plus 1 μM α,β-methylene ATP for 30 min and then again following washout of phentolamine and α,β-methylene ATP. Calibration bars are 1 mN (vertical) and 10 min (horizontal). Drug applications are indicated. *B*. Histogram showing the mean amplitudes of contractile responses to 10 μM linopirdine and 1 μM XE991 under control conditions (C) and following 30 min exposure to 10 μM phentolamine (Phen) or Phen plus 1 μM α,β-methylene ATP (α,β-mATP). Responses were measured in each vessel as a percentage of the contractile response to 50 mM KCl. The number of observations is shown next to each bar.

Linopirdine is known to evoke the release of neurotransmitters by inhibiting neuronal K^+ ^channels [14]. It is therefore possible that KCNQ channel blockers constrict pulmonary arteries by releasing neurotransmitters from nerve terminals in the vessel wall, which in turn activate the contractile response. The pulmonary circulation is innervated by sympathetic nerves, which release noradrenaline to activate contraction via α_1_-adrenoceptors located on the smooth muscle. To investigate the potential involvement of noradrenaline in the response to the KCNQ channel blockers, we determined the ability of phentolamine, an α-adrenoceptor antagonist, to prevent the constrictor response. As shown in Fig. 3, phentolamine (10 μM), at a concentration more than sufficient to inhibit the actions of nerve released noradrenaline, had no significant effect on the contractions induced by 10 μM linopirdine or 1 μM XE991. In rat small intrapulmonary arteries the main transmitter evoking excitation and contraction is ATP, acting on P2 receptors [17]. We investigated the potential role of this transmitter by blocking its action with α,β-methylene ATP (1 μM), applied in the presence of phentolamine (10 μM). At this concentration α,β-methylene ATP depolarises the membrane and causes contraction, followed by repolarisation and relaxation over the next 15–30 min as P2X1 receptors become desensitised [17,18]. At this time, vessels fail to respond to the application of ATP [18]. Following 30 min exposure to 1 μM α,β-methylene ATP, linopirdine (10 μM) and XE991 (1 μM) continued to induce constriction of rat pulmonary artery (Fig. 3C). The amplitudes of the contractions induced by linopirdine and XE991 were not significantly different when the drugs were applied under control conditions or after 30 min exposure to α,β-methylene ATP and phentolamine (Fig. 3D).

### Pulmonary artery constriction requires voltage-dependent Ca2+ influx

**Figure 5:**
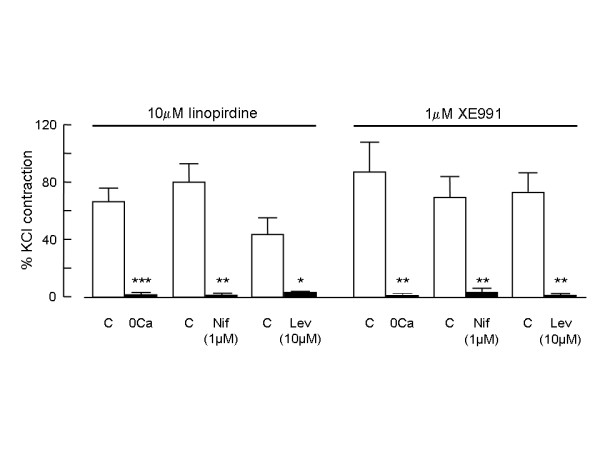
**Contraction requires voltage-dependent Ca^2+ ^influx**. Histogram comparing the mean amplitudes of contractile responses to 10 μM linopirdine or 1 μM XE991 under control conditions and in the presence of Ca^2+^-free (0Ca) bathing solution (n = 6 for both drugs), 1 μM nifedipine (Nif; n = 5 for both drugs) or 10 μM levcromakalim (Lev; n = 4 for linopirdine; n = 5 for XE991). Responses were measured in each vessel as a percentage of the contractile response to 50 mM KCl. ***p < 0.001, **p < 0.01, *p < 0.05 compared with paired control.

To determine how much of the contraction induced by KCNQ channel blockers required Ca^2+ ^influx from the extracellular space, we investigated the effect on the response of removing extracellular Ca^2+^. When vessels were exposed to Ca^2+^-free PSS for 20 min before applying 10 μM linopirdine (Fig. 4A) or 1 μM XE991 (Fig. 4B) the drugs had essentially no effect on tension, although they produced contraction in the same vessels exposed to normal PSS, whether applied before the test in Ca^2+^-free solution or after the normal Ca^2+ ^concentration had been restored. Fig. 5 summarises the mean results and shows that in the absence of extracellular Ca^2+ ^the contractile responses to linopirdine and XE991 were essentially abolished.

Several distinct pathways could potentially mediate Ca^2+ ^influx underlying the contraction of pulmonary artery smooth muscle. To determine the contribution of voltage-gated, L-type Ca^2+ ^channels, we investigated the effects of the selective Ca^2+ ^channel antagonist, nifedipine, on contractions induced by the KCNQ channel blockers. When nifedipine (1 μM) was applied to vessels pre-constricted with 10 μM linopirdine (Fig. 4C) or 1 μM XE991 (Fig. 4D) the tension rapidly declined to the baseline. In addition, when vessels were pre-exposed to nifedipine (1 μM) for 10 min, linopirdine and XE991 failed to produce contraction (not shown). Comparison of the mean amplitudes of contractions evoked by linopirdine or XE991 in the absence and presence of nifedipine shows that nifedipine essentially abolished the contractile responses to both drugs (Fig. 5).

If the contractions induced by linopirdine and XE991 depend on membrane depolarisation and activation of Ca^2+ ^influx through voltage-gated Ca^2+ ^channels, then they should be inhibited under conditions that promote hyperpolarisation and oppose the depolarising action of the drugs. To determine if this was the case, we examined how the contractions were affected by the K^+ ^channel opening drug, levcromakalim. This drug activates K_ATP _channels and causes hyperpolarisation of pulmonary artery smooth muscle cells [19]. At 10 μM, levcromakalim hyperpolarises pulmonary artery smooth muscle cells by around 15 mV [20], taking the membrane potential well away from the voltage threshold for activating L-type Ca^2+ ^channels. When applied in the presence of 10 μM levcromakalim neither 10 μM linopirdine (Fig. 4E) nor 1 μM XE991 (Fig. 4F) caused contraction in vessels that responded well to both drugs after levcromakalim was washed from the tissue with PSS for around 10 min. Comparison of the mean amplitudes of contractions evoked by linopirdine or XE991 in the absence and presence of levcromakalim shows that the contractile responses to both drugs were essentially abolished (Fig. 5).

## Discussion

The KCNQ channel blockers, linopirdine and XE991, were both found to act as potent constrictors of rat and mouse pulmonary arteries. Linopirdine constricted arteries from both species with an EC_50 _of around 1 μM, which is similar to the 2–3 μM concentrations found for 50% inhibition (IC_50_) of native M current in hippocampal neurons and sympathetic ganglia [15,21]. It is also close to the IC_50 _values reported for inhibition of homomeric KCNQ1 (8.9 μM), KCNQ2 (~4 μM) and KCNQ3 (4.8 μM) channels and heteromeric KCNQ2/3 channels (3.5–10 μM) and at least 10-fold lower than reported for other homomeric or heteromeric KCNQ channels [2]. XE991 generally has a 10-fold lower IC_50 _for KCNQ channels than linopirdine, although the relative potency of the drugs varies among different KCNQ subunits and subunit combinations [2]. It blocks KCNQ1 and KCNQ2 homomers, as well as KCNQ2/3 heteromers, with an IC_50 _of 0.6–0.8 μM, which is similar to the EC_50 _estimated for pulmonary artery constriction. Our EC_50 _measurements may have been affected by the presence of a relaxant action at the top end of the concentration-effect curve, leading to a possible underestimate. Nevertheless, the potencies found in this study are among the highest reported for linopirdine or XE991 actions in any tissue, consistent with the idea that they produced pulmonary vasoconstriction through an interaction with KCNQ channels.

The pulmonary vasoconstrictor effects of linopirdine and XE991 appear to be mediated by a direct action on the smooth muscle cells of the vessel wall. They do not require the presence of an intact endothelium, because removing the endothelium did not alter the contractile response to either drug. Since KCNQ channel blockers are known to stimulate neurotransmitter release [14], it was important to check the potential involvement of neuronal KCNQ channels in the vasoconstrictor response, the blockade of which might result in transmitters being released from nerve endings in the artery wall. Neither inhibition of α_1_-adrenoceptors with phentolamine, nor desensitization of P2X receptors with α,β-methylene ATP, had any effect on the pulmonary vasoconstrictor responses to linopirdine or XE99l. Since these are the main transmitters mediating electrical and contractile responses to nerve stimulation in rat intrapulmonary arteries [17,22], it is highly unlikely that neurotransmitters released in the vessel wall play a significant role in mediating the pulmonary vasoconstrictor effect of the KCNQ channel blockers.

The pulmonary vasoconstriction induced by linopirdine or XE991 was abolished in Ca^2+^-free medium. This indicates that the contraction was dependent upon Ca^2+ ^influx from the extracellular space into the smooth muscle cells and did not involve Ca^2+ ^release from intracellular stores. Furthermore, abolition of the constrictor responses in the presence of nifedipine indicates that Ca^2+ ^entered the cells through voltage-gated, L-type Ca^2+ ^channels. This is what would be expected if the KCNQ channel blockers were producing constriction by blocking resting K^+ ^channels and inducing depolarisation of the smooth muscle cell membrane. That this is the mechanism underlying the vasoconstrictor responses to linopirdine and XE991 is further supported by the ability of levcromakalim, a K_ATP _channel opener, to abolish the responses. At the concentration used, levcromakalim hyperpolarizes pulmonary artery smooth muscle cells close to the K^+ ^equilibrium potential [19,20]. This is demonstrated by its lack of effect on the tension developed by vessels exposed to a high extracellular K^+ ^concentration [20,23], where vessels contract due to membrane depolarization and Ca^2+ ^influx. The pronounced effect of levcromakalim on the responses to linopirdine and XE991 contrasts with only partial inhibition of the constrictor responses to a number of receptor agonists, where it blocks only the component of contraction that results from voltage-dependent Ca^2+ ^influx [20,23]. This implies that the constrictor responses to linopirdine and XE991 are strongly dependent on the membrane potential and/or K^+ ^flux, and voltage-gated Ca^2+ ^influx.

Linopirdine has generally been found to be well tolerated in humans, with few adverse effects. Pulmonary vasoconstriction is, however, a potentially serious side effect that might not be picked up by routine examinations. Since linopirdine and XE991 constricted pulmonary arteries while having no effect on mesenteric arteries from the same species, they are likely to elevate pulmonary arterial pressure without a change in systemic blood pressure. Consistent with this, linopirdine had no effect on blood pressure or pulse rate in human volunteers [24]. Possible effects on the pulmonary circulation have not been investigated *in vivo*. The lack of effect on mesenteric arteries suggests that the vasoconstrictor action of KCNQ channel blockers may be selective for the pulmonary circulation. A recent report indicates that linopirdine (50 μM) and XE991 (10 μM) can also modulate the spontaneous contractions of portal vein [13], but these effects were observed at higher concentrations than required to constrict pulmonary artery. Further studies are required to determine if other blood vessels can be affected by these drugs.

## Conclusion

The KCNQ K^+ ^channel blocking drugs, linopirdine and XE991, are potent and powerful constrictors of pulmonary arteries. This is a potentially serious side effect of the drugs and should be considered as KCNQ blockers are developed for clinical use. The lack of a similar response in mesenteric arteries suggests that this action may be selective for the pulmonary circulation. All of the data point to an action on the smooth muscle cells of pulmonary arteries, involving K^+^-channel blockade, membrane depolarisation and Ca^2+ ^influx as the mechanism underlying the response. This strongly suggests that KCNQ channels play a functional role in pulmonary artery smooth muscle, most likely contributing to the resting K^+ ^conductance and resting potential. If so, then drugs like retigabine, which activate KCNQ channels [2], could be effective and selective dilators of pulmonary arteries and may prove useful in the treatment of pulmonary hypertension.

## Competing interests

The author(s) declare that they have no competing interests.

## Authors' contributions

SJ carried out most of the experimental work, contributed to the design and coordination of the study and helped to draft the manuscript. PB carried out some of the experimental work. AMG conceived of the study, participated in its design and coordination and drafted the manuscript. All authors read and approved the final manuscript.
